# Seek the Simillimum

**Published:** 1883-01

**Authors:** Carroll Dunham


### HOMŒOPATHIC PHYSICIAN,

A MONTHLY JOURNAL OF MEDICAL SCIENCE.

"If our school ever gives up the strict inductive method of Hahnemann, we are lost, and deserve only to be mentioned as a caricature, in the history of medicine."—CONSTANTINE HERING.

Vol. III. JANUARY, 1883. No. 1.

### SEEK THE SIMILLIMUM.

It is amazing that the idea of using one specific as a substitute for another specific could ever be entertained, since the virtues of a specific reside in its peculiar individual properties, which are never common to two different substances. Nevertheless, even at the present day and in our latest works on materia medica we find the subject of the substitution of Arsenic for Quinine gravely discussed, and statistics referred to to show, as the case may be, its inferiority or superiority to Quinine. As might be supposed, the testimony of different physicians differs widely on this point. Some affirm that almost all the cases treated by them during a certain period were promptly cured by Arsenic while they proved rebellious to Quinine. Others succeeded with Arsenic in a smaller proportion of cases and in a larger with Quinine; while others, again, found Arsenic of comparative little use, Quinine curing nearly every case. Finally, others, again, failed with Arsenic and Quinine alike, but succeeded with other drugs less often used, as Ipecacuanha, or Eupatorium, or Nux vomica.

Now, it is a wonderful thing that medical men should still argue, in the face of these statistics, upon a question of the relative value of certain drugs in the treatment of a disease regarded not in the light of the individuals affected by it, but solely with reference to its great pathological features. It seems to me the only sound deductions from these testimonies are these: That there are diversities in the form in which intermittent fever appears in different persons and in different epidemics; that these forms require different remedies, and that thus there is a form which is capable of being cured by Arsenic, and by nothing else; a form capable of being cured by Quinine, and nothing else; and so of other drugs. In this view, when a case of intermittent fever presents itself, the question can never be: Is Arsenic a better remedy for this disease than Quinine is? Does it offer greater chances of a cure? There can be no better or worse. The question is between right and wrong; suitable and not suitable. The question would be always: Which remedy corresponds to this particular case, and is, therefore, indicated in it?—CARROLL DUNHAM.

